# Modified ligation procedure for prolapsed haemorrhoids *versus* stapled haemorrhoidectomy for the management of symptomatic haemorrhoids (MoLish): randomized clinical trial

**DOI:** 10.1093/bjsopen/zrac064

**Published:** 2022-05-13

**Authors:** Haibo Yang, Zhan Shi, Wei Chen, Teng Chen, Peilin Ding, Jandong Wang, Jiazhi Gao

**Affiliations:** Departments of Anorectal Surgery, Putuo Hospital, Shanghai University of Traditional Chinese Medicine, Shanghai, P.R. China; Departments of Anorectal Surgery, Putuo Hospital, Shanghai University of Traditional Chinese Medicine, Shanghai, P.R. China; Departments of Anorectal Surgery, Putuo Hospital, Shanghai University of Traditional Chinese Medicine, Shanghai, P.R. China; Departments of General Surgery, Putuo Hospital, Shanghai University of Traditional Chinese Medicine, Shanghai, P.R. China; Departments of Anorectal Surgery, Putuo Hospital, Shanghai University of Traditional Chinese Medicine, Shanghai, P.R. China; Departments of Anorectal Surgery, Putuo Hospital, Shanghai University of Traditional Chinese Medicine, Shanghai, P.R. China; Departments of Anorectal Surgery, Putuo Hospital, Shanghai University of Traditional Chinese Medicine, Shanghai, P.R. China

## Abstract

**Background:**

The aim of this study was to compare a modified ligation procedure *versus* stapled haemorrhoidectomy (SH) in patients with symptomatic haemorrhoids.

**Methods:**

This randomized trial included patients with symptomatic haemorrhoids treated in Shanghai from May 2018 to September 2021. Eligible patients were randomly 1:1 assigned the modified ligation procedure for prolapsed haemorrhoids (MLPPH) and SH groups. The primary outcome was the assessment of efficacy at 6 months after the intervention. The operating time, incidence of complications, clinical effectiveness (pain, Wexner incontinence, haemorrhoid symptom severity (HSS) scores, and 6-month cure rate) were collected, and quality-adjusted life years (QALYs) were adopted as indicator for the cost-effectiveness analysis (CEA).

**Results:**

Out of 187 patients screened, 133 patients were randomized (67 for MLPPH and 66 for SH). One patient in the MLPPH group was excluded, and two patients were lost to follow-up. The mean operating time was longer in MLPPH than in SH (57.42 min *versus* 30.68 min; *P* < 0.001). The median pain score was higher in SH than in MLPPH at postoperative day 3 (*P* = 0.018), day 7(*P* = 0.013), and day 14 (*P* = 0.003). The median Wexner incontinence score was higher in SH than in MLPPH at postoperative month 1 (*P* = 0.036) and month 3 (*P* = 0.035), but was similar in the two groups at month 6. In addition, the median HSS score was lower in MLPPH than in SH 6 months after surgery (*P* = 0.003). The 6-month cure rate was higher in MLPPH than in SH (*P* = 0.003). CEA showed lower mean costs in MLPPH than in SH (EUR 1080.24 *versus* EUR 1657.97; *P* < 0.001) but there was no significant difference in effectiveness (*P* = 0.181). However, MLPPH was cost-effective (incremental cost-effectiveness ratio, −120 656.19 EUR/QALYs).

**Conclusion:**

MLPPH was documented as a longer but cost-effective procedure, it provided lower short-term pain, and Wexner and HSS scores.

**Registration number:** Chinese Clinical Trial Registry ChiCTR1800015928 (http://www.chictr.org.cn/searchproj.aspx).

## Teaser

The aim of this study was to compare clinical efficacy and to perform a cost-effectiveness analysis to define the possible benefits of the modified ligation procedure *versus* stapled haemorrhoidectomy in patients with symptomatic haemorrhoids.

## Introduction

Haemorrhoidal disease is one of the most common and frequently occurring benign anorectal disorders. With the progression of its severity, the prolapse of the anal cushions and the rectal mucosa gradually worsen. The clinical manifestations are the prolapse of an anal mass, pain, rectal bleeding and pruritus, anal swelling, rectum emptying and defecation difficulties, anal obstruction, and other symptoms^1^. Excision of the redundant rectal mucosa and suspension of anal cushions are the main methods to treat prolapsed haemorrhoids^2^; however it is difficult to completely resect the redundant rectal mucosa in a narrow rectal cavity 4.5 cm away from the margin^3,4^. Stapled haemorrhoidectomy (SH) is a technique for the treatment of haemorrhoids by reducing the mucosa and haemorrhoidal prolapse with a circular suturing device based on the sliding anal lining theory. SH has a slightly higher recurrence rate, but patients return to normal activity more quickly after SH than after a traditional haemorrhoidectomy^5^. After SH for haemorrhoids, annular anastomosis might result in annular scarring and stapler nail residue, which will lead to postoperative complications, such as anastomotic stenosis or anal swelling, and these can seriously affect the recovery of patients^6^. An alternative treatment is the tissue-selecting technique, which is a novel SH technique that targets haemorrhoids, leaves the uninvolved mucosal bridge intact and avoids circumferential annular anastomosis^7^.

To reduce tissue trauma, some surgeons use automatic haemorrhoid ligation devices to perform rubber banding or elastic thread ligation at the base of the haemorrhoid. Blocking the blood supply of the ligated tissue can produce ischaemia, atrophy, and necrosis during the removal of haemorrhoid tissue; these techniques mainly include rubber band ligation (RBL) and automatic elastic thread ligation (ATH) for haemorrhoids. Mild bleeding, pain, vasovagal symptoms, band slippage, dysuria, anal fissures, and chronic longitudinal ulcers are generally considered the most frequent minor complications. Massive bleeding, haemorrhoid thrombosis, severe pain, urinary retention requiring catheterization, pelvic sepsis, and death are major complications that are uncommonly reported^8^. The RBL technique for haemorrhoids was modified by our group into a modified ligation procedure for prolapsed haemorrhoids (MLPPH). This randomized clinical trial aimed to explore the safety and cost-effectiveness of MLPPH compared with SH in the management of symptomatic haemorrhoids.

## Methods

This randomized, single-blind, and single-centre clinical trial of MLPPH *versus* SH was conducted at Putuo Hospital Affiliated with Shanghai University of Traditional Chinese Medicine from May 2018 to September 2021. The study was a parallel-group trial with a 1:1 allocation ratio. The same team of eight anorectal surgeons performed both types of anal cushion suspensions.

The study followed the Ethical Principles for Medical Research Involving Human Subjects as outlined in the Declaration of Helsinki. The study protocol was approved by the Ethics Committee of Putuo Hospital Affiliated with Shanghai University of Traditional Chinese Medicine on 30 January 2018 (approval number: PTEC-A-2018-2-1), and registered with both the Chinese Clinical Trial Registry on 4 May 2018 (http://www.chictr.org.cn/searchproj.aspx, ChiCTR1800015928), and the National Health Information Guarantee Platform.

### Inclusion and exclusion criteria

**Fig. 1:**
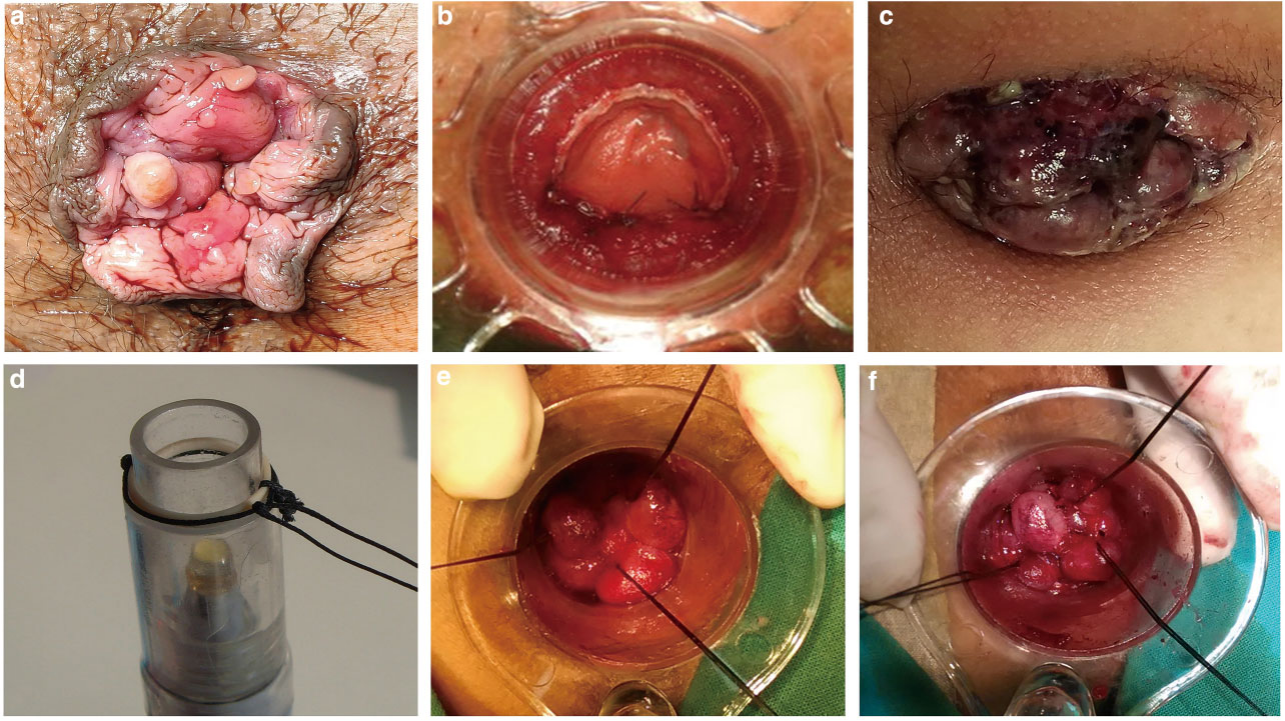
**Case diagrams**  **of the treatment of prolapsed haemorrhoids**

Eligible participants were patients aged 18–80 years with symptomatic second- to fourth-degree haemorrhoids (*Fig. 1*). All participants voluntarily agreed to participate in the study and provided written informed consent. Patients were excluded if they had acute haemorrhoidal oedema, infection or bleeding; had inflammatory bowel disease, or acute or chronic diarrhoea; had an anal fistula, perianal sepsis, colorectal malignancy, complete rectal prolapse, or perianal dermatosis; had a history of surgery for haemorrhoids, a pre-existing sphincteric injury, or symptomatic incontinence; had diabetes, anaemia, malnutrition, or immunodeficiency; had serious heart, liver, or kidney disease, or blood coagulation dysfunction; were pregnant or menstruating; or were unable to provide informed consent.

**a** Symptomatic haemorrhoid. **b** After stapled haemorrhoidectomy for haemorrhoids, annular anastomosis might result in annular scarring and stapler nail residue. **c** After rubber band ligation for haemorrhoids, the rubber band detached too soon, and the anus was swollen. **d–f** Case diagram of the modified ligation procedure for prolapsed haemorrhoids (MLPPH). **d** Placement of the rubber band and silk loop. **e** Ligation, suspension, and fixation at 3–4 sites in the lower area of the rectum 3.5–4.5 cm away from the anal margin. **f** Ligation at the area of the upper haemorrhoid pole 1.5–2.0 cm from the anal margin.

### Randomization, allocation concealment, and blinding

**Fig. 2:**
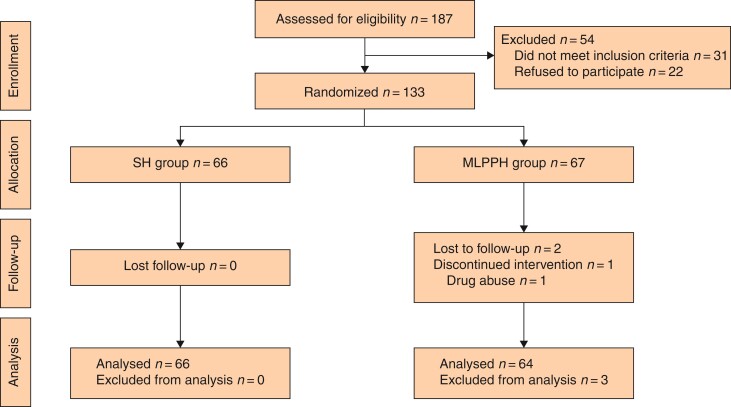
CONSORT diagram of the trial

Eligible patients were included in the study according to the selection criteria. The randomization algorithm was stratified by haemorrhoid grade. After determining haemorrhoid grade, the participants were individually randomly assigned to undergo either the MLPPH or SH (at a 1:1 ratio) (*Fig. 2*). The randomized assignments were produced by a statistician, without any involvement of the surgical team, by way of a computerized random number generator (SPSS^®^ version 19; IBM, Armonk, New York, USA). Blinding was achieved with opaque sealed envelopes containing allocations. To determine the trial arm designation, the envelopes were sent to each attending physician and opened by study staff, in sequence, once participant consent was obtained. To avoid any possible response bias, research staff with whom the participant had no previous contact administered the final questionnaire and efficacy assessment. This study was open-label with no blinding of participants, clinicians, or research staff.

The study was conducted at Putuo Hospital Affiliated with Shanghai University of Traditional Chinese Medicine from May 2018 to September 2021. SH, stapled haemorrhoidectomy; MLPPH, modified ligation procedure for prolapsed haemorrhoids.

### Procedures

The trial design refers to both the Hubble and eTHOS trials^9,10^. Preoperative data collected before the procedure included age, sex, disease duration, co-morbidities, haemorrhoid grade, and previous haemorrhoid treatments. Before surgery, the colon was prepared with oral polyethylene glycol electrolyte powder (Shutaiqing; Staidson Biopharmaceuticals Co., Beijing, China) in all patients. The patients did not undergo mechanical colon preparation or preoperative prophylaxis with oral antibiotics.

Eight surgeons were involved in the study. Each operation was performed by two experienced anorectal surgeons. After intraspinal anaesthesia, the patients were placed in a folding knife position by two experienced anorectal surgeons for the procedure. Intravenous cephalosporin and metronidazole were used for perioperative antibiotic prophylaxis.

The MLPPH was performed with a device (Zhongren Biomedical Technology Co., Shanghai, China) that applies a rubber band and a silk loop to each ligation site via a proctoscope. The method for placing the rubber band and silk loop is shown in *Fig. 1*. The proctoscope was placed at the lower area of the rectum 3.5–4.5 cm away from the anal margin, the redundant rectal mucosa was isolated with a device, and the rubber band and silk loop were released onto the lesion at the same time. The rectal mucosal tissue was bound, and then the silk loop was tightened to form a double ligation. The above procedure was completed at 3–4 sites. The proctoscope was removed, and the same procedure was performed in the area of the upper haemorrhoid pole 1.5–2.0 cm from the anal margin. The specific location and number of ligation points were determined according to the shape, size, and position of the patient’s haemorrhoids. This band ligation constricts the blood supply, causing the haemorrhoids to become ischaemic before being sloughed off ∼1–2 weeks later. The resultant fibrosis reduces the likelihood of prolapse. The procedure is a basic surgical skill that all attending physicians within the department are familiar with and are competent in performing.

SH was performed with a PAH32 stapler (Brightness Medical Device Co., Changzhou, Jiangsu, China). A 36-mm circular dilator was first introduced, and then 2-0 Safil sutures (B. Braun Surgical, S.A., Rubi, Spain) were inserted at the submucosal layer at least 2 cm above the dentate line, *Fig. 1*. A 32-mm procedure for prolapse and haemorrhoids (PPH) stapler was then introduced into the rectum, and the ends of the purse-string suture were removed from the side hole of the stapler. Traction was maintained on the purse-string suture such that a substantial amount of mucosal tissue was engaged by the stapler. The stapler was closed tightly and then fired. After removal of the stapler gun, haemostatic 2-0 Safil absorbable sutures were placed at any site of bleeding.

After surgery, oral paracetamol, tramadol hydrochloride tablets, and intramuscular ketorolac trometamol were prescribed for pain control as required. Patients were discharged home when there was no wound bleeding, fever, or difficulty defecating and when the wound pain was significantly relieved.

Questionnaires for the quality of life were given to the participants; the data were collected over the telephone, WeChat, or handed in at the 1-month visit.

Complementary, adjunctive treatments (such as dietary counselling, stool hygiene and habits, consumption of fibre, and use of local therapies such as red-light therapy) were not specifically included in the trial and were prescribed at the surgeon’s discretion and along the lines of the pragmatic study design.

### Outcome measures

The primary outcome was the 6-month cure rate following SH or the MLPPH. Based on the definitions of the Traditional Chinese Medicine (TCM) Industry Standard (Standard for Diagnosis and Efficacy of Anorectal Diseases in TCM) issued by the People's Republic of China in 1995 and the ‘Guideline for Diagnosis and Treatment of Anorectal Diseases in TCM’ issued by the Anorectal Branch of the China Association of TCM in 2012, combined with clinical practice, the efficacy standards applied at 6 months after surgery were as follows: cure was defined by the disappearance of haemorrhoid symptoms (haematochezia, prolapse, pain) and by normal anal appearance and function; improvement was defined by the relief of haemorrhoid symptoms (haematochezia, prolapse, pain) and improved anal appearance and function.

Cure rate = cured cases/total cases × 100 per cent

The secondary outcomes aimed to identify which treatment (MLPPH or SH) is the most cost-effective, the least painful with the fewest complications, and has the greatest effect on the patient’s quality of life. Therefore, the secondary outcomes were as follows: persistence of symptoms, the symptom severity score, the health-related quality of life (HRQoL) score, Wexner incontinence score, postoperative pain, complications, operative time, clinical appearance of haemorrhoids on proctoscopy, and healthcare costs/cost-effectiveness/QALYs^11–14^.

Briefly, the persistence of significant symptoms was measured at the 6-month follow-up. The symptom severity score was adapted from Nyström *et al*^13^. This score was the sum of the scores from all five questions and was therefore a number on a nominal scale ranging from 0 to 15, with a greater number indicating more severe symptoms. The haemorrhoid symptom severity (HSS) score was determined before randomization and at 1, 3, and 6 months after surgery. The HRQoL score was determined with the EQ-5D™ 3L^11^ (EuroQoL Group, Rotterdam, The Netherlands). A summary index with a maximum score of 1 was derived from the five dimensions by converting the score using a table of scores. The maximum score of 1 on this scale indicated the best health state, in contrast with the scores of the individual questions, where higher scores indicated more severe or frequent problems. EQ-5D-3L was measured before randomization and at 1, 7, 21 days and 3, 6 months after surgery. Continence determined using the validated Wexner incontinence score^14^. The Wexner incontinence score was simply the sum of the scores from all five questions and was also a number on a nominal scale ranging from 0–20, with a greater number indicating more severe incontinence. The Wexner incontinence score was measured before randomization and at 1, 3, and 6 months after surgery. Pain was determined using a visual analogue scale (VAS) 10-scale VAS12, for which ‘0’ indicates ‘no pain’, and ‘10’ indicates the ‘worst imaginable pain’ The VAS pain score was observed after the operation and at postoperative days 1, 3, 7, and 14. Surgical complications were measured 1 month after operation. The clinical appearance of haemorrhoids was assessed on proctoscopy following 6 months of symptom persistence. Finally, a healthcare costs/cost-effectiveness/QALYs was conducted.

Data were collected to conduct a full economic evaluation. The healthcare system perspective was used in this study, so only the direct medical costs were recorded as costs. The direct medical costs included expenses for hospitalization, diagnosis, instruments, examinations, laboratory examinations, nursing care, treatments, drugs, surgical materials (including costs for surgery and materials), and other materials (including haemorrhoid ligation devices and PPH staplers). Direct non-medical costs, indirect costs and intangible costs were excluded from this study. The expenses for the instruments, examinations, and laboratory examinations that were direct medical costs were not included in the cost analysis because some patients had a relevant examination before admission, and the examination of patients and the patients’ individual requirements were greatly different, which had a great influence on the results. The cost considered in this study was defined as the total expenses for hospitalization, diagnosis, nursing care, treatments, drugs, surgical materials, and other materials.

The cost–utility analysis was performed in terms of the incremental cost per QALY of the MLPPH *versus* SH over the 6-month follow-up interval. Patients were asked to complete the EQ-5D-3L at baseline and after the treatment. The EQ-5D-3L descriptive system describes the general health of patients in terms of five dimensions: mobility, self-care, usual activities, pain/discomfort, and anxiety/depression. Each dimension has three levels (no problems, some or moderate problems, and extreme problems), resulting in a total of 243 unique healthy states. According to the Japanese time trade-off values, the health utility value was generated, which represented the health status of the interviewees. The QALYs of the patients were assessed with the area under the curve method^9^.

Cost-effectiveness analysis (CEA) was used as the main method of economic evaluation, the outcome of which is expressed as the incremental cost-effectiveness ratio (ICER). The ICER indicates the cost increase required for each additional unit of effectiveness and was adopted as the decision-making index. Additionally, a one-way sensitivity analysis and probabilistic sensitivity analysis were performed to determine any further sources of uncertainty.

### Statistical analysis and health economic analysis

The primary outcome (6-month cure rate), was used for sample size calculations. An equivalence trial must show that the true absolute difference between two proportions is no greater than a prespecified, clinically meaningful value (▵); any difference equal to or less than ▵ is considered clinically unimportant. The value for type I error was specified at 5 per cent (α = 0.05) in a bilateral approximation with a minimum power of 90 per cent (0.10 probability of type II error). To demonstrate equivalence with (1 − α) *×* 100 per cent confidence, it is sufficient to produce a (1 − α) *×* 100 per cent confidence interval (c.i.) that is completely captured in the equivalence interval (−▵, ▵). A sample size of 60 per group was calculated, such that for a value of ▵ = 0.23 and a true difference of zero, a 95 per cent confidence interval would fall completely within the equivalence interval with a probability of 0.90. It was planned that 64 patients would be enrolled in each group to allow for violations of the protocol and/or non-evaluable patients. The value of ▵ = 0.23 was based on the difference in the 6-month cure rate between the historical MLPPH cure rate (91 per cent) in the authors’ department and the SH cure rate (68 per cent) in articles published before 2018^15^.

Statistical analysis was performed on all available data before the operation and at the end of the study intervention. There were no missing data. The Kolmogorov–Smirnov test was used to test for normality. Categorical variables, presented as numbers with percentages, were analysed using the chi-squared test or Fisher’s exact test. Continuous data are presented as the mean and s.d. Intergroup comparisons were carried out using the independent-samples *t* test when the data approximated a normal distribution. Data that did not conform to a normal distribution, such as the course of disease, duration of hospital stay, HSS score, and Wexner score, were expressed as the median and interquartile range (i.q.r.). The Mann–Whitney *U* test was used for intergroup comparisons. All hypothesis tests were two-tailed, with α = 0.10 for normality tests and α = 0.05 for other hypothesis tests; *P* < 0.05 was considered statistically significant. SPSS® version 19 (IBM, Armonk, New York, USA) was used for the statistical analysis.

Using a decision-tree model as the model structure, we evaluated the outcome between the MLPPH and SH in the treatment of prolapsed haemorrhoids and the economics of the quality of life and hospital costs. The model consisted of two branches that were divided into two branches from the decision node, representing the intervention strategies of the MLPPH and SH on prolapsed haemorrhoids. After each intervention strategy, the chance node was divided into two branches, representing cure and improvement. The model time span was 6 months and is shown in *Fig. S1*.

TreeAge Pro 2011 (TreeAge Software, Williamstown, Massachusetts, USA) was used for the cost–utility analysis. Because there are many assumptions in the model, one-way sensitivity analysis was conducted to evaluate the robustness of the base-case results and to address the uncertainty by varying costs up to 20 per cent in each direction. A Monte Carlo simulation was used to conduct the probability sensitivity analysis on the parameter uncertainties. After 1000 Monte Carlo simulations, the cost-effectiveness acceptability curve (CEAC) was determined to graph the per cent change of iterations for which strategy is cost-effective. All cost parameters adopted a γ distribution, and the utility value parameters adopted a β distribution.

## Results

Between 1 May 2018 and 30 September 2021, 133 participants (of the 187 screened) were randomly assigned to undergo the MLPPH or SH. Sixty-six participants were allocated to undergo SH, and 67 were allocated to undergo MLPPH. Three of these participants (both randomly assigned to the MLPPH group) were removed from the trial due to ineligibility: one patient had a history of drug abuse and weakened response to analgesic drugs, and two patients were lost to follow-up. Finally, 64 patients were included in the MLPPH group (*Fig. 2*). Primary outcome data were available for 130 participants (64 in the MLPPH group and 66 in the SH group). At 6 months, 130 fully completed patient questionnaires were returned. Patient follow-up was completed on 15 October 2021. The preoperative characteristics were similar in the two groups (*Table 1*). There were no differences in age, sex, disease course, or haemorrhoid grade between the two groups. The mean operating time was longer in the MLPPH group than in the SH group (57.42 min *versus* 30.68 min; *P* < 0.001).

**Table 1:** Preoperative characteristics of participants in the two groups

	SH (*n* = 66)	MLPPH (*n* = 64)	P*
**Sex**			0.991
Male	31 (47.0)	30 (46.9)	
Female	35 (53.0)	34 (53.1)	
**Age (years)**†	53.11 (13.33)	49.05 (14.61)	0.100§
**Disease course (months)**‡	48 (12–120)	18 (6–117)	0.116¶
**Haemorrhoid grade**			0.987
II	16 (24.2)	16 (25.0)	
III	36 (54.5)	34 (53.1)	
IV	14 (21.2)	14 (21.9)	

SH, stapled haemorrhoidectomy; MLPPH, modified ligation procedure for prolapsed haemorrhoids. Values are n (%) unless indicated otherwise; *Chi-squared or Fisher’s exact test, except. †Median(range). ‡Mean(s.d.). §Student’s t test. ¶Mann–Whitney *U* test.

The breakdown of postoperative complications in the two groups is shown in *Table 2*. There were six cases of anastomotic haematoma in the SH group but no cases of perianal haematoma/thrombosis in the MLPPH group. The incidence of urinary retention and anal distension was higher in the SH group than in the MLPPH group, but there was no significant difference between the two groups. The incidence of postoperative cutaneous bridge oedema was significantly higher in the MLPPH group than in the SH group (32.8 *versus* 6.1 per cent; *P* < 0.001). There was no difference in the total incidence of postoperative complications between the two groups.

**Table 2:** Postoperative complications

Complications	SH (*n* = 66)	MLPPH (*n* = 64)	OR (95% c.i.)	P*
**Perianal haematoma/thrombosis**	6 (9.1)	0		
**Cutaneous bridge oedema**	4 (6.1)	21 (32.8)	7.57 (2.42 to 23.62)	<0.001
**Urinary retention**	20 (30.3)	17 (26.6)	0.83 (0.39 to 1.79)	0.637
**Anal distension**	36 (54.5)	24 (37.5)	0.50 (0.25 to 1.01)	0.051
**Total incidence of complications**	40 (60.6)	40 (62.5)	1.08 (0.53 to 2.20)	0.824

Values are n (%) unless indicated otherwise. SH, stapled haemorrhoidectomy; MLPPH, modified ligation procedure for prolapsed haemorrhoids. *Chi-squared or Fisher’s exact test.

The HSS score gradually decreased after surgery in both groups. The HSS score was similar in the two groups at postoperative months 1 and 3. However, the HSS score was lower in the MLPPH group than in the SH group at 6 months after surgery (median (range), 0 (0–1) *versus* 1 (0–3); *P* = 0.003), as shown in *Table 3*. The sub-analysis for the HSS scores in patients with grade III and grade IV haemorrhoids showed that the HSS score was also lower in the MLPPH group than in the SH group at 6 months after surgery (median (range), 0 (0–1) *versus* 1 (0–3); less than 0.001), as shown in *Table 4*.

**Table 3:** Comparison of HSS scores

Follow-up time point	SH (*n* = 66)	MLPPH (*n* = 64)	P*
**Scores before surgery**	6 (4–7)	6 (5–7)	0.733
**Scores 1 month after surgery**	2 (1–4)	2 (1–3.8)	0.285
**Scores 3 months after surgery**	1 (0–3)	1 (0–2)	0.127
**Scores 6 months after surgery**	1 (0–3)	0 (0–1)	0.003

Values are median (range). HSS, haemorrhoid symptom severity; SH, stapled haemorrhoidectomy; MLPPH, modified ligation procedure for prolapsed haemorrhoids. P*, SH *versus* the MLPPH; *Mann–Whitney *U* test.

**Table 4:** Sub-analysis for the HSS scores in patients with grade III and grade IV haemorrhoids

Follow-up time point	SH (*n* = 50)	MLPPH (*n* = 48)	P*
**Scores before surgery**	7 (6–8)	7 (5–8)	0.515
**Scores 1 month after surgery**	2 (1–4)	2 (1–3)	0.125
**Scores 3 months after surgery**	2 (0–3)	1 (0–2)	0.054
**Scores 6 months after surgery**	1 (0–3)	0 (0–1)	<0.001

Values are median (range). HSS, haemorrhoid symptom severity; SH, stapled haemorrhoidectomy; MLPPH, modified ligation procedure for prolapsed haemorrhoids. P*, SH *versus* the MLPPH; *Mann–Whitney *U* test.

**Fig. 3:**
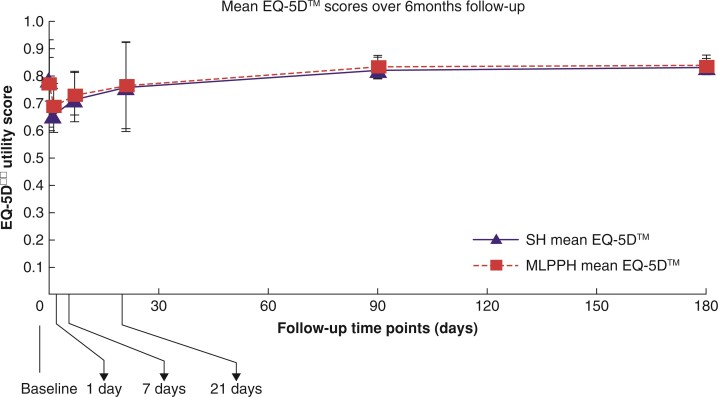
Mean EQ-5D™ scores with 95% c.i. over the 6-month follow-up

Before intervention, the mean(s.d.) health utility (EQ-5D-3L) was 0.79(0.08) in the SH group and was 0.77(0.16) in the MLPPH group (*P* = 0.519). The mean health utility in both groups declined at postoperative day 1 and then gradually increased. The mean health utility was lower in the SH group than in the MLPPH group on postoperative day 1 (mean(s.d.), 0.65(0.10) *versus* 0.69(0.06); *P* = 0.006). The mean health state in both groups returned to above the baseline values by postoperative month 3. The mean health utility scores in the groups were nearly similar, with no significant differences between the two groups, at postoperative days 7 and 21 and postoperative months 3 and 6 (*Fig. 3*).

The mean health utility was lower in the SH group than in the MLPPH group at postoperative day 1. The mean health utility was similar, with no significant differences between the two groups at postoperative days 7 and 21 and at postoperative months 3 and 6. SH, stapled haemorrhoidectomy; MLPPH, modified ligation procedure for prolapsed haemorrhoids.

The Wexner incontinence score gradually decreased in both groups over time. The Wexner incontinence score was lower in the MLPPH group than in the SH group at postoperative month 1 (median (range), 0 (0–2) *versus* 1 (0–3); *P* = 0.036) and month 3 (median (range), 0 (0–1) *versus* 0 (0–2); *P* = 0.035); however, the Wexner incontinence score was similar in the two groups at postoperative month 6, as shown in *Table 5*.

**Table 5:** Comparison of Wexner incontinence scores

Follow-up time point	SH (*n* = 66)	MLPPH (*n* = 64)	P*
**Score before surgery**	3 (1.75–6.25)	3 (1–4.75)	0.143
**Score 1 month after surgery**	1 (0–3)	0 (0–2)	0.036
**Score 3 months after surgery**	0 (0–2)	0 (0–10	0.035
**Score 6 months after surgery**	0 (0–1)	0 (0–0)	0.160

Values are median (range). SH, stapled haemorrhoidectomy; MLPPH, modified ligation procedure for prolapsed haemorrhoids. P*, SH *versus* the MLPPH group. *Mann–Whitney *U* test.

Patients rated their current pain due to haemorrhoids after the operation and at four time points over the subsequent 14 postoperative days using a 10-point VAS. The VAS score in both groups decreased over time. SH was associated with more short-term pain than the MLPPH, as shown in *Table 6*. The pain score was similar in the groups at postoperative day 1 (median (range), 4 (3–5.3) *versus* 3 (3–4); *P* = 0.277); it was higher in the SH group than in the MLPPH group at postoperative day 3 (median (range), 3 (2–5) *versus* 3 (2–3); *P* = 0.018), day 7 (median (range), 2 (1–3) *versus* 1 (1–2); *P* = 0.013), and day 14 (median (range), 1 (0–2) *versus* 1 (0–1); *P* = 0.003).

**Table 6:** Comparison of VAS pain scores

Follow-up time point	SH (*n* = 66)	MLPPH (*n* = 64)	P*
**Score after surgery**	5 (3–6.5)	4.5 (4–6)	0.924
**Score 1 day after surgery**	4 (3–5.3)	3 (3–4)	0.277
**Score 3 days after surgery**	3 (2–5)	3 (2–3)	0.018
**Score 7 days after surgery**	2 (1–3)	1 (1–2)	0.013
**Score 14 days after surgery**	1 (0–2)	1 (0–1)	0.003

Values are median (range). SH, stapled haemorrhoidectomy; MLPPH, modified ligation procedure for prolapsed haemorrhoids. P*, SH *versus* the MLPPH. *Mann–Whitney *U* test.

Further analysis for the assessment of clinical efficacy at 6 months after the interventions was performed. The number of participants with a cure at 6 months was 46 (69.7 per cent) in the SH group compared with 58 (90.6 per cent) in the MLPPH group. The 6-month cure rate was higher in the MLPPH group than in the SH group (chi-squared = 8.894, 1 d.f.; *P* = 0.003), as shown in *Table 7*.

**Table 7:** Primary outcomes

	SH (*n* = 66)	MLPPH (*n* = 64)	Difference (%)	P*
**Cured**	46 (69.7)	58 (90.6)	20.9	0.003
**Improvement**	20 (30.3)	6 (9.4)	−20.9	

Values are n (%) unless indicated otherwise. SH, stapled haemorrhoidectomy; MLPPH, modified ligation procedure for prolapsed haemorrhoids. *Chi-squared or Fisher’s exact test.

### Cost-effectiveness analysis

**Fig. 4:**
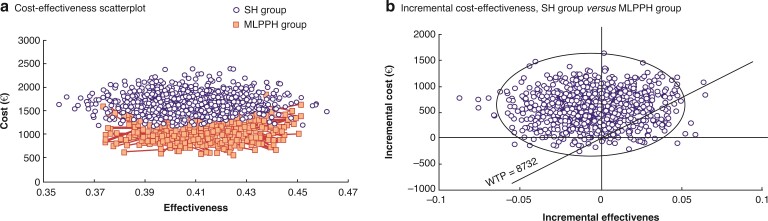
**a** Cost-effectiveness scatter plot for the two surgical procedures. The CER was lower for the MLPPH group than for SH. **b** Incremental cost-effectiveness scatterplot for the two surgical procedures. At the 8732 EUR/QALYs gained threshold, SH may be more costly and more effective (upper right quadrant) and more costly and less effective (upper left). MLPPH, modified ligation procedure for prolapsed haemorrhoids; SH, stapled haemorrhoidectomy; CER, cost-effectiveness ratio; QALY, quality-adjusted life year.

The main findings of the within-trial CEA suggest that the MLPPH seemed to be cost-effective compared with SH. The mean (s.d.) total cost per patient for the MLPPH was EUR 1080.24 (248.01) compared with EUR 1657.97 (279.92) for SH. In the base-case analysis, the difference in the mean total cost was EUR 577.73 lower for the MLPPH than for SH, the number of QALYs was higher for the MLPPH than for SH, and the cost-effectiveness ratio was lower for the MLPPH than for SH (*Fig. 4*), resulting in an ICER of −120 656.19 EUR/QALYs, as shown in *Table 8*. The sub-analysis for the CEA in patients with grade III and grade IV haemorrhoids showed that the MLPPH also seemed to be cost-effective compared with SH. The mean (s.d.) total cost per patient for the MLPPH was EUR 1147.33 (247.55) compared with EUR 1690.28 (290.32)for SH. In the base-case analysis, the difference in the mean total cost was EUR 603.74 lower for the MLPPH than for SH, the number of QALYs was higher for the MLPPH than for SH, and the cost-effectiveness ratio was lower for the MLPPH than for SH, resulting in an ICER of −126 650.09 EUR/QALYs, as shown in *Table 9*. These findings indicate that the MLPPH could not only achieve the same health benefits but also save costs compared with SH and has the advantage of cost-effectiveness. At the 8732 EUR/QALY gained threshold, SH may be more costly and more effective (upper right quadrant) or more costly and less effective (upper left) (*Fig. 4*).

**Table 8:** CEA analysis

	SH (*n* = 66)	MLPPH (*n* = 64)	Difference	P*
**Cost (EUR)**	1657.97 (279.72)	1080.24 (248.01)	**−**577.73	<0.001
**Effectiveness (QALYs)**	0.40766 (0.02307)	0.41245 (0.01691)	0.00479	0.181
**CER (EUR/QALY)**	4086.68 (757.36)	2629.05 (637.51)	−1457.63	<0.001
**ICER (EUR/QALY)** −120656.19				

Values in parentheses are the mean(s.d.) unless indicated otherwise. CEA, cost-effectiveness analysis; CER, cost-effectiveness ratio; ICER, incremental cost-effectiveness ratio; QALY, quality-adjusted life year; SH, stapled haemorrhoidectomy; MLPPH, modified ligation procedure for prolapsed haemorrhoids. *Student’s *t* test.

**Table 9:** Sub-analysis for the CEA in patients with grade III and grade IV haemorrhoids

	SH (*n* = 50)	MLPPH (*n* = 48)	Difference	P*
**Cost (EUR)**	1690.28 (290.32)	1147.33 (247.55)	−603.74	<0.001
**Effectiveness (QALYs)**	0.40738 (0.02303)	0.41167 (0.01840)	0.00429	0.312
**CER (EUR/QALY)**	4168.03 (781.95)	2797.29 (637.13)	−1370.74	<0.001
**ICER (EUR/QALY)** −120656.19				

Values in parentheses are the mean(s.d.) unless indicated otherwise. CEA, cost-effectiveness analysis; CER, cost-effectiveness ratio; ICER, incremental cost-effectiveness ratio; QALY, quality-adjusted life year; SH, stapled haemorrhoidectomy; MLPPH, modified ligation procedure for prolapsed haemorrhoids. *Student’s *t* test.

**Fig. 5:**
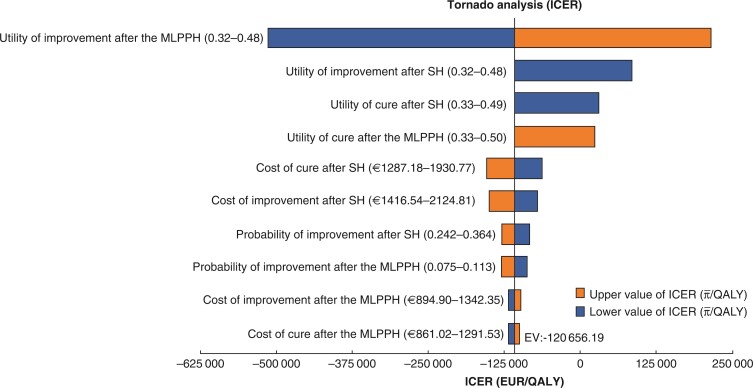
**ICER tornado diagram for one-way sensitivity analysis for the MLPPH *versus* SH** The vertical line denotes the base-case expected value (−120 656.19 EUR/QALYs). The utility of improvement after the MLPPH was the most sensitive parameter. The cost of improvement after the MLPPH and the cost of cure after the MLPPH were the least influential on the results of the model. MLPPH, modified ligation procedure for prolapsed haemorrhoids; SH, stapled haemorrhoidectomy; ICER, incremental cost-effectiveness ratio; QALY, quality-adjusted life year.

The results of the one-way sensitivity analyses are shown in *Fig. 5*. In order of most to least influential, the variables were as follows: utility of improvement after the MLPPH (estimate, 0.32–0.48), utility of improvement after SH (estimate, 0.32–0.48), utility of cure after SH (estimate, 0.33–0.49), utility of cure after the MLPPH (estimate, 0.33–0.50), cost of cure after SH (estimate, EUR 1287.18–1930.77), cost of improvement after SH (estimate, EUR 1416.54–2124.81), probability of improvement after SH (estimate, 0.242–0.364), probability of improvement after the MLPPH (estimate, 0.075–0.113), cost of improvement after the MLPPH (estimate, EUR 894.90–1342.35), and cost of cure after the MLPPH (estimate, EUR 861.02–1291.53). The ICER was not substantially changed by changes in any of the above parameters.

**Fig. 6:**
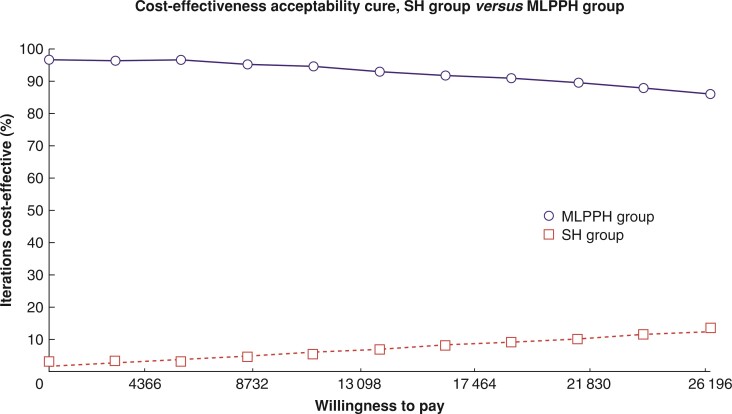
**CEAC for the probabilistic sensitivity analysis of the MLPPH *versus* SH** Regardless of how the WTP changed, the MLPPH was more likely to be more economical than SH for the treatment of symptomatic haemorrhoids, which supports the cost-effectiveness advantage of the MLPPH. MLPPH, modified ligation procedure for prolapsed haemorrhoids; SH, stapled haemorrhoidectomy; CEAC, cost-effectiveness acceptability curve; WTP, willingness to pay.

The probability sensitivity analysis assumed that the cost variation followed the γ distribution and that the utility value variation followed the β distribution. We put each parameter into the model and drew the CEAC after 1000 Monte Carlo simulations. The results showed that, regardless of how the willingness to pay (WTP) changed, the MLPPH was more likely to be economical than SH for prolapsed haemorrhoids, which shows the cost-effectiveness advantage of the MLPPH, as shown in *Fig. 6*.

## Discussion

Prolapsed haemorrhoids are a common clinical condition. When haemorrhoids progress to grade III–IV, the abnormalities in both the physiological function and pathological anatomy of the rectum are substantial^16,17^ and the effect of non-surgical medical treatment is poor^18^, and surgical treatment is often required^19^. In 1975, Thomson proposed the sliding anal lining theory, that is, the theory that the anal canal is lined by specialized, highly vascular cushions of submucosal tissue that provide substantial support. An irregular bowel habit is likely to be associated with hard and bulky stools, which cause straining and a greater likelihood of the cushions being pushed out of the anal canal. Furthermore, straining during defecation, by producing a general increase in the venous pressure, may cause engorgement of the cushions during defaecation, making their displacement more likely. Treitz’s muscle might become stretched and disrupted if repeatedly subjected to such forces, which could cause intermittent and then permanent prolapse of the cushions^20^. In 1993, Antonio Longo introduced a novel technique for the treatment of severe haemorrhoids by reducing mucosal and haemorrhoidal prolapse with a circular suturing device based on the above theory, and in 1998, introduced the use of circular suturing devices for its treatment^21^.

Generally, low-grade internal haemorrhoids can be effectively treated with medication and nonoperative measures (such as RBL and injection sclerotherapy). The ligation procedure is generally not appropriate for high-grade internal haemorrhoids or for failure of non-surgical approaches or complications^22^. In the past 10 years, the MLPPH was adopted by our group to treat low-grade and high-grade haemorrhoids by reducing mucosal and haemorrhoidal prolapse based on the above theory.

The results of the present study showed that the clinical application of the MLPPH was safe and reliable, and no serious clinical adverse events occurred after the operation. SH was associated with more short-term pain than MLPPH. The mean pain score was higher in the SH group than in the MLPPH group during the early postoperative phase. In this study, the Wexner incontinence score was higher in the SH group than in the MLPPH group at postoperative months 1–3; however, the Wexner incontinence score was similar in the two groups at postoperative month 6. In addition, the mean HSS score was lower in the MLPPH group than in the SH group at 6 months after surgery, although the operating time was longer in the MLPPH group than in the SH group.

At present, studies on the above surgical schemes for haemorrhoids are mainly focused on safety and effectiveness, and there is a lack of economic evaluation for the above surgical schemes. A CEA was used to evaluate the effectiveness and economics of the MLPPH *versus* SH in this study. In the base-case analysis, the costs were lower in the MLPPH group than in the SH group. Intervention for haemorrhoids is essentially aimed at improving the patient’s quality of life, which therefore becomes an important indicator of success. These results suggest that the majority of patients in both groups had an improvement in their quality of life above baseline from postoperative month 3 onwards after the interventions. Although no long-term difference was observed between the two groups, both interventions did result in a small improvement in the quality-of-life scores; therefore, both interventions seem worthwhile from this perspective. The results showed that the MLPPH for haemorrhoids was cost-effective, indicating that the MLPPH could not only achieve the same health benefits but also save costs compared with SH and has the advantage of cost-effectiveness.

The results of the one-way sensitivity analyses showed that there were 10 variables that influenced the results of the CEA, which were as follows, in order of most to least influential: utility of improvement after the MLPPH, utility of improvement after SH, utility of cure after SH, utility of cure after the MLPPH, cost of cure after SH, cost of improvement after SH, probability of improvement after SH, probability of improvement after the MLPPH, cost of improvement after the MLPPH, and cost of cure after the MLPPH. The ICER was not substantially changed by changes in the above parameters. The probability sensitivity analysis assumed that the cost variation followed the γ distribution and that the utility value variation followed the β distribution. Each parameter was included in the model, and the CEAC was drawn after 1000 Monte Carlo simulations. The results showed that, regardless of how the WTP changed, the MLPPH was more likely to be economical than SH for prolapsed haemorrhoids, which shows the cost-effectiveness advantage of the MLPPH.

However, this study also has potential limitations. First, the trial experienced few issues in enrolling patients over an interval of more than 3 years. The main reason for non-inclusion was related to patient consent for inclusion. Second, the length of follow-up might not be adequate. Third, there is a lack of an international clinical efficacy evaluation system for haemorrhoids. Fourth, this study included a small sample size, and the decision-tree model assumes that the outcomes are independent, which may affect the analysis results. Fifth, currently, there have been no studies on the utility value preference of patients with symptomatic haemorrhoids, and the effectiveness value in this study had a large uncertainty, which may cause a certain bias in the study results. Despite the above limitations, the results of the base-case analysis in this study are consistent with those of the sensitivity analysis.

Finally, the MLPPH was documented as a cost-effective approach, although it was a longer procedure. It provided lower short-term pain and lower Wexner and haemorrhoid severity scores and achieved a better clinical therapeutic effect than SH in the treatment of symptomatic haemorrhoids.

## Supplementary Material

zrac064_Supplementary_Data

## Data Availability

All data included in this study are available upon request by contact with the corresponding author.
