# Supplementary figures and images for: Modified ligation procedure for prolapsed haemorrhoids versus stapled haemorrhoidectomy for the management of symptomatic haemorrhoids (MoLish): randomized clinical trial
Source: BJS Open. 2022 May 13;6(3):zrac064. doi: 10.1093/bjsopen/zrac064 (PMC9099087; doi:10.1093/bjsopen/zrac064)

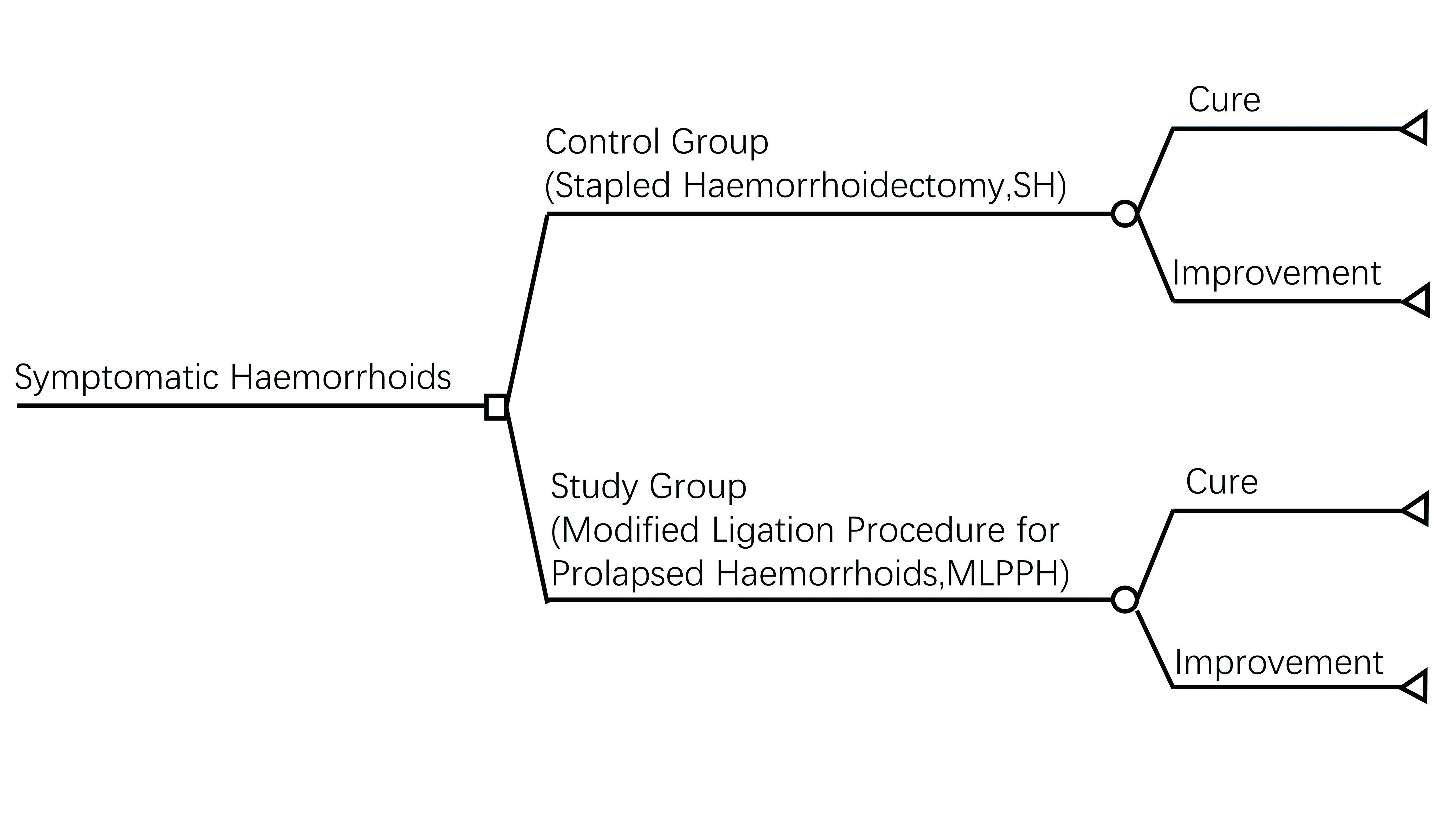

Supplement: zrac064_Supplementary_Data [file zrac064_supplementary_data.zip › Supplementary_Figure_1.jpg]
